# Regulation on Calcium Oxalate Crystallization and Protection on HK-2 Cells of Tea Polysaccharides with Different Molecular Weights

**DOI:** 10.1155/2020/5057123

**Published:** 2020-05-12

**Authors:** Hong Liu, Xin-Yuan Sun, Feng-Xin Wang, Jian-Ming Ouyang

**Affiliations:** ^1^Department of Chemistry, Institute of Biomineralization and Lithiasis Research, Jinan University, Guangzhou 510632, China; ^2^Department of Urology, Guangzhou Institute of Urology, Guangdong Key Laboratory of Urology, The First Affiliated Hospital of Guangzhou Medical University, Guangzhou Medical University, Guangzhou, Guangdong 510230, China

## Abstract

The regulation on calcium oxalate (CaOx) crystallization and protective effect on human proximal tubular epithelial cells (HK-2) of four green tea polysaccharides (TPSs) with molecular weights of 10.88 (TPS0), 8.16 (TPS1), 4.82 (TPS2), and 2.3 kDa (TPS3) were comparatively studied. XRD, Fourier transform infrared spectroscopy, and scanning electron microscopy results revealed that TPS1, TPS2, and TPS3 can increase the percentage of the dihydrate crystalline phase in CaOx crystals and reduce the size of CaOx monohydrate crystals. TPSs increased the absolute value of the zeta potential of CaOx crystal and inhibited crystal nucleation and aggregation. The nucleation inhibition rates of TPS1, TPS2, and TPS3 to CaOx crystallization were 56.67%, 75.52%, and 52.92%, respectively, and their aggregation inhibition rates were 22.34%, 47.59%, and 21.59%, respectively. TPS preprotection can alleviate the oxidative damage of HK-2 cells caused by oxalate, increase cell viability, protect cell morphology, and reduce lactate dehydrogenase release and reactive oxygen species levels. The degraded TSPs, especially TPS2 with moderate molecular weight, may be used as a green drug to inhibit stone formation.

## 1. Introduction

Kidney stone is one of the most common diseases, and its main components are calcium oxalate (CaOx) monohydrate (COM) and CaOx dihydrate (COD) [1], and COM is more common than COD [2]. Stone formation is generally dependent on the level of imbalance between the urinary inhibitors and promoters of crystallization [3]. Many stone inhibitors, such as organic macromolecular glycosaminoglycans, citric acid, and phosphate, are present in urine [4, 5].

In patients with kidney stones, oxalate and CaOx crystals induce the generation of free radicals in renal epithelial cells, which generate oxidative stress, thereby leading to the formation of kidney stones [6]. Plant polysaccharides not only can reduce cell oxidative damage by scavenging free radicals [7] but also can inhibit the nucleation, aggregation, and growth of crystals [8, 9]. The biological activity of plant polysaccharides is related to the physicochemical properties of the polysaccharide, such as the molecular weight of polysaccharides and acidic groups in polysaccharides [10]. Shi et al. [11] showed that the degraded polysaccharide with a low molecular weight (44 kDa) from *Enteromorpha prolifera* has a stronger antioxidant capacity than crude polysaccharide from *E. prolifera* (1400 kDa). In vivo, low-molecular-weight polysaccharides have better protective effects on oxalate-induced oxidative damage than high-molecular-weight heparin and fucoidan [12, 13].

Tea is one of the most extensive drinks in the world, especially in Asia. Tea polysaccharide (TPS) is one of the most important components in tea. Although many studies have been conducted on TPSs, most of them focused on pharmacological properties, which include anti-inflammatory, anticancer, antioxidation, hypocholesterolemic, blood pressure lowering, and other biological activities [14–16], and the research on inhibiting CaOx kidney stones are few.

In our previous study [17], we used H_2_O_2_ to degrade the original TPS (TPS0) with a molecular weight of 10.88 kDa and obtained three degraded polysaccharides with molecular weights of 8.16 (TPS1), 4.82 (TPS2), and 2.31 (TPS3) kDa, and their structures were characterized. TPSs have an antioxidant effect and repair effect on damaged human renal proximal tubular cells (HK-2). After TPS repair, the damage of COM to HK-2 cells can be effectively reduced, and the adhesion of COM crystals to cells can be reduced [18].

In this study, we studied the regulation of TPSs on CaOx crystallization and the ability of TPSs to protect renal epithelial cells from oxidative damage to develop new green, natural antistone drugs.

## 2. Materials and Methods

### 2.1. Materials and Apparatus

#### 2.1.1. Materials

Green tea polysaccharide (TPS) was purchased from Shaanxi Ciyuan Biological Co., Ltd. Three different molecular weights of degraded polysaccharides TPS1, TPS2, and TPS3 were obtained by H_2_O_2_ oxidative degradation according to Reference [16]. The molecular weight and carboxyl (–COOH) content of TPSs are shown in Table 1. Human proximal tubular epithelial cells (HK-2) were purchased from Shanghai cell bank of the Chinese Academy of Sciences (Shanghai, China).

The cell proliferation assay kit (CCK-8), lactate dehydrogenase (LDH) kit, hematoxylin and eosin (HE) staining kit, and reactive oxygen species assay kit (DCFH-DA) were from Shanghai Beyotime Bio-Tech Co., Ltd., Shanghai, China. Fetal bovine serum and cell culture medium (DMEM) were from Gibco, USA. Penicillin and streptomycin were from Beijing Pubo Biotechnology Co., Ltd., Beijing, China. Cell culture plates of 6, 12, and 96 well were from NEST, China. Anhydrous calcium chloride (CaCl_2_), sodium oxalate (Na_2_Ox), and the other conventional reagents were from Guangzhou chemical reagent factory, China.

#### 2.1.2. Apparatus

Fourier transform infrared spectroscopy (FT-IR) was from EQUINOX55, Bruker, Germany. Ultraviolet-visible spectrophotometer was from Cary 500, Varian company, USA. The X-ray powder diffractometer (D/MAX2400) was from Japan. The X-L type environmental scanning electron microscope (ESEM) was from Philips, Eindhoven, Netherlands, and a Nano Measurer 1.2.5 Software (Fudan University, China) was used to determine the average size of the CaOx crystals from the SEM images. Inverted fluorescence microscope was from Olympus company, Japan. The optical microscope was from OlyMPUS, CKX41, Japan. The Zetasizer Nano ZS90 apparatus was from Malvern, England. The flow cytometer was from Beckman, Gallios, USA. The microplate reader was from SafireZ, Tecan, Switzerland.

### 2.2. Experimental Methods

#### 2.2.1. Crystallization of CaOx

Experiments were carried out with slight modification according to Reference [19]. The reaction was maintained and conducted in a 100 mL beaker at 37°C. Firstly, 4.4 mg, 6.6 mg, 13.2 mg, 22.0 mg, and 35.2 mg of TPSs with different molecular weights were added to 22 mmol/L CaCl_2_ solution (20 mL) and magnetically stirred for 10 min. Sodium oxalate (22 mmol/L, 20 mL) was then added to the mixture until the final volume of 44 mL was obtained. The final concentrations of both Ca^2+^ and Ox^2−^ were 10 mmol/L, and the polysaccharide concentrations were 0.1, 0.15, 0.3, 0.5, and 0.8 g/L. After stirring for 10 min, the mixture was maintained at 37°C for 2 h, and the suspension was centrifuged to obtain CaOx deposits. The bottom deposits of CaOx were dried for component analysis through XRD and FT-IR, and some precipitates were ultrasonically dispersed with anhydrous alcohol to facilitate the morphological observation through scanning electron microscopy (SEM). The relative percentage contents of COM and COD in the CaOx precipitates were calculated through the *K* value method and according to the XRD patterns:

(1) $$\begin{array}{c} \text{COD}\%=\frac{I_{\text{COD}}}{I_{\text{COD}}+I_{\text{COM}}}, \end{array}$$

where *I*_COM_ and *I*_COD_ are the intensity of the main diffraction peak ($\bar{1}$01) crystal plane of COM and the main diffraction peak (200) crystal plane of COD, respectively.

#### 2.2.2. Zeta Potential Measurement of CaOx Crystals

CaOx crystals were dispersed in double distilled water with 200 *μ*g/mL concentration. After ultrasonication for 10 min, the zeta potential was detected with a Zetasizer Nano ZS90 apparatus at 25°C.

#### 2.2.3. CaOx Crystallization Assay

The change in turbidity in the reaction system was detected with a UV spectrophotometer, and the inhibition effect of TPSs on the nucleation (*S*_N_) and aggregation rates (*S*_A_) of the CaOx crystals were studied [20]. CaOx crystallization was achieved by using a mixture of CaCl_2_ solution (8 mmol/L) and sodium oxalate (1 mmol/L), containing 200 mmol/L NaCl, and 10 mmol/L NaAc and by adjusting the pH to 5.7.

A total of 1 mL of CaCl_2_ solution (8 mmol/L) were stirred constantly at 37°C in the absence or presence of TPSs (0.1 g/L). Crystallization was induced by adding 1 mL of Na_2_Ox solution (1 mmol/L) to reach final concentrations of 4 mmol/L CaCl_2_ and 0.5 mmol/L Na_2_Ox. The change in turbidity over 20 min was measured at 620 nm.

The following three parameters characterize the crystallization process [21]: first, crystal *S*_N_, which is the maximum increase in optical density (OD) over time and mainly reflects the maximum rate of formation of new particles, thereby representing crystal nucleation; second, *S*_A_, which is derived from the maximum decrease in OD [22]; and third, maximum time (*t*_max_), at which the crystals can neither nucleate nor grow [23]. All three parameters are measurable in the crystallization process of CaOx.

The percentage inhibition was calculated from the nucleation and *S*_A_, as follows: [1 − (*S*_NT_/*S*_NC_)] × 100 for the rate of nucleation and [(1 − *S*_AT_/*S*_AC_)] × 100 for the rate of aggregation, where *S*_NT_ and S_AT_ are the slopes in the presence of the TPSs, and *S*_NC_ and *S*_AC_ are the slopes of the control experiment.

#### 2.2.4. Cell Culture

HK-2 cells were cultured in DMEM medium containing 10% fetal bovine serum in a 5% CO_2_ humidified environment at 37°C. Upon reaching a monolayer of 80%–90% confluence, cells were gently blown after trypsinization to form a cell suspension for subsequent cell experiments.

#### 2.2.5. Toxicity Detection of TPSs on HK-2 Cells

Cell suspension with a cell concentration of 1 × 10^5^ cells/mL was inoculated per well in 96-well plates and incubated for 24 h. Afterward, the culture medium was removed, and 100 *μ*L of 20, 60, and 100 *μ*g/mL TPSs with various molecular weights was added, and each concentration was repeated in five parallel wells. After incubation for 24 h, CCK-8 was added to each well and incubated for 1.5 h. Absorbance (*A*) was measured at 450 nm according to the CCK-8 kit instruction. Cell viability was determined using the following equation:

(2) $$\begin{array}{c} \text{Cell viability}\left(\%\right)=\frac{A\left(\text{treatment group}\right)}{A\left(\text{control group}\right)}\times 100 \end{array}$$

#### 2.2.6. Protective Effect of TPSs on HK-2 Cells by CCK-8

Cell suspension with a cell concentration of 1 × 10^5^ cells/mL was inoculated per well in 96-well plates and incubated for 24 h. The cells were divided into three groups: (1) the control group, where only serum-free DMEM culture medium was added; (2) the protection group, where serum-free medium containing TPSs with concentrations of 20, 40, 60, 80 and 100 *μ*g/mL was added, and the culture medium was aspirated after 12 h. The cells were then treated with 2.8 mmol/L sodium oxalate dissolved in PBS and incubated for 3.5 h; and (3) the injured group, in which 2.8 mmol/L sodium oxalate dissolved in PBS was added and incubated for 3.5 h. The absorbance values were measured using the enzyme mark instrument at 450 nm to detect the protective capacity of TPSs.

#### 2.2.7. Lactate Dehydrogenase (LDH) Release Assay

Cell suspension with a cell concentration of 1 × 10^5^ cells/mL was inoculated per well in 96-well plates and incubated for 24 h. The cells were divided into three groups: (1) the control group, where only serum-free DMEM culture medium was added; (2) protection group, where serum-free medium containing TPSs with concentrations 80 *μ*g/mL was added, and the culture medium was aspirated after 12 h; then, the cells were treated with 2.8 mmol/L sodium oxalate dissolved in PBS and incubated for 3.5 h; and (3) the injured group, in which 2.8 mmol/L sodium oxalate dissolved in PBS was added and incubated for 3.5 h. LDH release was measured with a microplate reader in accordance with the LDH kit test instructions. Absorbance was measured at 490 nm with a reference wavelength of 620 nm.

#### 2.2.8. Cell Morphology Observation by Hematoxylin-Eosin (HE) Staining

The density of seeded cells and experimental grouping was the same as those in Section 2.2.7. After the treatment time was reached, the supernatant was then removed by aspiration and the cells were washed twice with PBS. Cells were fixed with 4% paraformaldehyde for 15 min and stained with hematoxylin and eosin according to the manufacturer's instructions. Morphological changes of the cells were observed under a microscope.

#### 2.2.9. Changes in Intracellular Reactive Oxygen Species (ROS) Levels

The density of seeded cells and experimental grouping was the same as those in Section 2.2.7. After the treatment time was reached, 500 *μ*L DCFH-DA diluted with serum-free culture medium at 1 : 1000 was added and incubated for 30 min at 37°C. ROS distribution was observed under a fluorescent microscope; the fluorescence intensity of intracellular ROS was quantitatively detected by flow cytometer.

#### 2.2.10. Statistical Analysis

Experimental data were expressed as the mean ± standard deviation ($\bar{x}\pm \text{SD}$). The experimental results were analyzed statistically using SPSS 13.0 software (SPSS Inc., Chicago, IL, USA). The differences in the means between the experimental groups and the control group were analyzed using one-way ANOVA, followed by the Tukey post hoc test. If *p* < 0.05, there was significant difference; if *p* < 0.01, the difference was extremely significant; and if *p* > 0.05, there was no significant difference.

## 3. Results

### 3.1. Degraded TPSs Induce COD Formation

**Figure 1:**
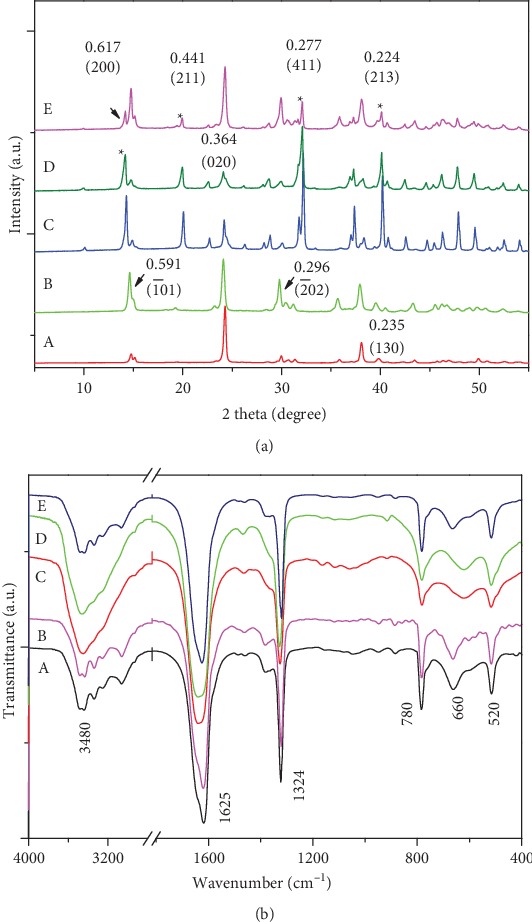
XRD patterns (a) and FT-IR spectra (b) of CaOx crystals induced by TPSs with different molecular weights. (A) Blank. (B) TPS0. (C)TPS1. (D) TPS2. (E) TPS3. *c*(Ca^2+^) = *c*(Ox^2−^) = 10 mmol/L; *c*(TPS): 0.1 g/L; crystallization time: 2 h; ∗ represents the main diffraction peaks of COD. The absence of ∗ represents the main diffraction peaks of COM.

Figure 1(a) showed the XRD spectra of CaOx crystals induced by TPSs with different molecular weights. The diffraction peaks that appeared at the spacing *d* of 0.591, 0.364, 0.296, and 0.235 nm were attributed to the ($\bar{1}$01), (020), ($\bar{2}$02), and (130) crystal planes of COM, respectively. The diffraction peaks at *d* of 0.617, 0.441, 0.277, and 0.224 nm were attributed to the (200), (211), (411), and (213) planes of COD, respectively. Figure 1(a) shows that TPS with different molecular weights induced COD formation in different proportions (Table 1). For example, when TPS concentration was 0.1 g/L, the percentages of TPS-induced COD crystals followed the order of TPS2 (80%)>TPS1 (73%)>TPS3 (31%)>TPS0 (0%); that is, TPS2 with moderate molecular weight had the highest induced COD content.

FT-IR spectra further confirmed XRD results. As shown in Figure 1(b), the FT-IR spectra of the CaOx crystals of the blank group, TPS0 (0.1 g/L), and TPS3 (0.1 g/L) were mainly the characteristic peaks of COM; that is, the formed crystals were mainly COM. However, in the presence of TPS1 (0.1 g/L) and TPS2 (0.1 g/L), the characteristic peaks of COD were the main peaks; that is, the formed crystals were mainly COD.

For COM crystals, a set of stretching vibration peaks was composed of five peaks at 3485–3047 cm^−1^ (Figure 1(b)), which belong to the O–H bond of the COM crystals. However, COD only has a single broad absorption peak in this region (Figure 1(b)) [24]. The appearances of the asymmetrical stretching *ν*_as_(COO−) and symmetrical stretching *ν*_s_(COO−) were near 1621 and 1325 cm^−1^, respectively, which indicated the presence of the COM crystals. However, the occurrence of *ν*_as_(COO−) and *ν*_s_(COO−) at approximately 1647 and 1326 cm^−1^ [25], respectively, indicated the presence of the COD crystals. In the fingerprint region, the absorption band of the COM crystals appeared at approximately 947, 885, 780, and 663 cm^−1^, while COD appeared at approximately 916 and 618 cm^−1^.

### 3.2. Effect of TPSs with Different Concentrations on COD Formation

**Figure 2:**
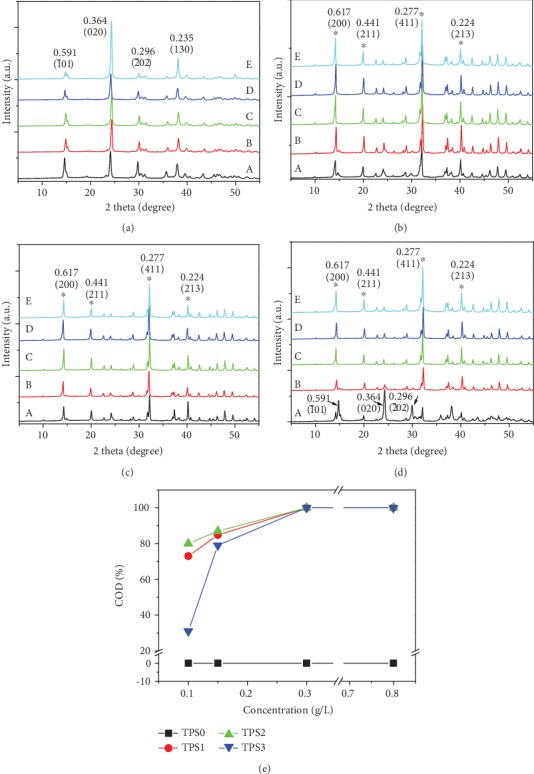
XRD patterns of CaOx crystals and COD content induced by different concentrations of TPSs. (a) TPS0. (b) TPS1. (c) TPS2. (d) TPS3. (e) Percentage of COD. *c*(TPS): (A) 0, (B) 0.1, (C) 0.15, (D) 0.3, (E) 0.8 g/L. *c*(Ca^2+^) = *c*(Ox^2−^) = 10 mmol/L; crystallization time: 2 h.

The XRD spectra of CaOx crystals induced by TPSs at different concentrations are shown in Figure 2. With the increase in TPS concentration, the diffraction peak of COD increased, thereby indicating that the percentage of induced COD increased. TPS1, TPS2, and TPS3 induced 100% COD at concentration of 0.3 g/L (Figure 2(e)). However, when the TPS0 concentration with the largest molecular weight was 0.8 g/L, the diffraction peak of COD was still not evident.

**Figure 3:**
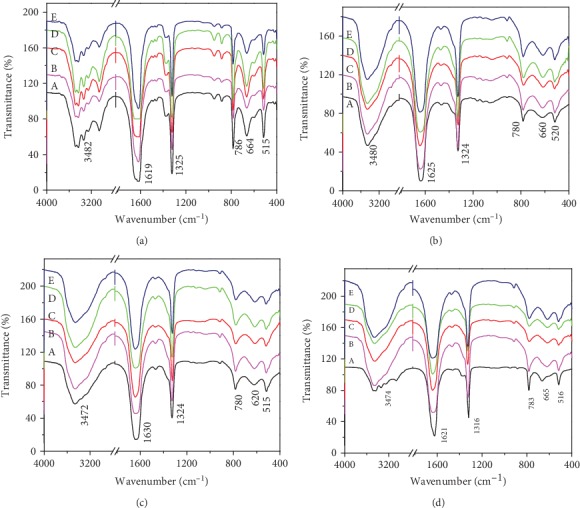
FT-IR spectra of CaOx crystals induced by different concentrations of TPSs. (a) TPS0. (b) TPS1. (c) TPS2. (d) TPS3. *c*(TPS): (A) 0, (B) 0.1, (C) 0.15, (D) 0.3, and (E) 0.8 g/L.

Figure 3 shows the FT-IR spectrum of the CaOx crystals induced by TPSs with different concentrations. With the increase in the TPS concentration from 0.1 g/L to 0.8 g/L, the *ν*_as_(COO−) and *ν*_s_(COO−) of the CaOx crystals underwent blue shifts at different degrees. For example, the *ν*_as_(COO−) in the crystals regulated by TPS2 increased from 1639 cm^−1^ to 1651 cm^−1^, and *ν*_s_(COO−) increased from 1323 cm^−1^ to 1342 cm^−1^ (Table 2). The results showed that the COM percentage gradually decreased, while COD gradually increased.

### 3.3. SEM Observation of CaOx Crystals Regulated by TPSs

**Figure 4:**
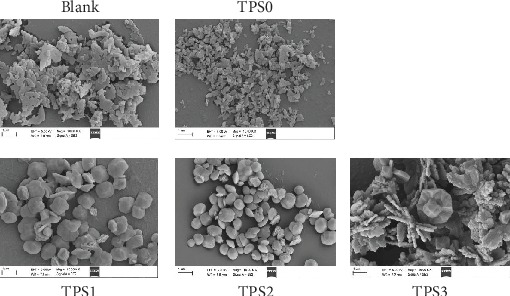
SEM images of CaOx crystals regulated by four TPSs. *c*(Ca^2+^) = *c*(Ox^2−^) = 10 mmol/L; *c*(TPS) = 0.1 g/L; crystallization time: 2 h.

Figure 4 shows the SEM images of the CaOx crystals induced by four TPSs at the concentration of 0.1 g/L. In the absence of polysaccharide, the formed crystals were COM crystals with irregular morphology and serious aggregation. After TPS0 was added, COM crystals were still obtained, but the crystal size was smaller than that of the control group. In the presence of 0.1 g/L TPS1 and TPS2, COD with blunt edges and corners were mainly formed, but the crystal size of the TPS2-regulated COD was small. TPS3-induced crystals had larger sizes, and the percentage of COD significantly decreased, and the degree of COM aggregation was also higher than that in TPS1 or TPS2.

In order to better quantify the difference in the particle size between different groups, we used Nano Measurer 1.2.5 Software (Fudan University, China) to determine the average size of the CaOx crystals from the SEM images. This size was a statistical result of the average data from 100 single particles. The average particle sizes of the crystals in the blank group and four TPS treatment groups are 1.81 ± 0.21 *μ*m, 1.02 ± 0.16 *μ*m (TPS0), 1.18 ± 0.28 *μ*m (TPS1), 0.88 ± 0.15 *μ*m (TPS2), and 2.64 ± 0.84 *μ*m (TPS3), respectively.

Figure 4 shows that the three degraded TPSs (TPS1, TPS2, and TPS3) can induce COD formation. TPS0 with the largest molecular weight cannot induce COD formation but can reduce the size and aggregation degree of the formed crystals. The percentage of the COD induced by TPS2 with moderate molecular weight was the largest, and the crystal size regulated by TPS2 was the smallest.

### 3.4. Effect of TPSs with Different Molecular Weights on Inhibition of CaOx Crystal Nucleation and Aggregation

**Figure 5:**
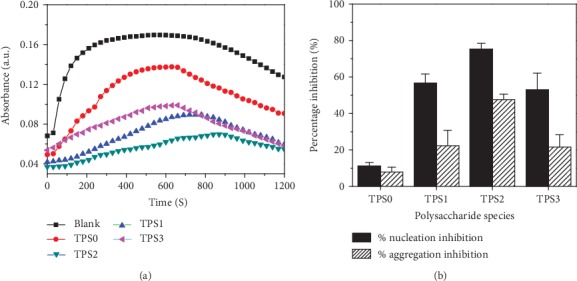
(a) Time-course measurements of OD_620_ in five control experiments at standard conditions (4 mmol/L CaCl_2_ and 0.5 mmol/L Na_2_Ox). (b) Percentage inhibition of calcium oxalate crystal nucleation and aggregation by four TPSs with different molecular weights. *c*(TPS) = 0.1 g/L.

Figure 5 shows the effect of four TPSs on nucleation and aggregation of the CaOx crystals. After TPSs with different molecular weights were added, the nucleation *t*_max_ of the crystal was prolonged (Table 3), and the *S*_N_ and the crystal *S*_A_ of CaOx crystal simultaneously reduced. A total of 0.1 g/L TPS0, TPS1, TPS2, and TPS3 resulted in the nucleation inhibition percentages of 11.12%, 56.67%, 75.52, and 52.92%, and crystal aggregation was inhibited by 7.84%, 22.34%, 47.59%, and 21.59%, respectively. Hence, the inhibition effect of TPSs on crystal nucleation and aggregation followed the order TPS2>TPS1>TPS3>TPS0, which indicated that TPS2 with a moderate molecular weight had the best inhibition effect on the nucleation and aggregation of the CaOx crystals.

### 3.5. Zeta Potential (*ζ*) of CaOx Crystals with TPSs Having Different Molecular Weights

**Figure 6:**
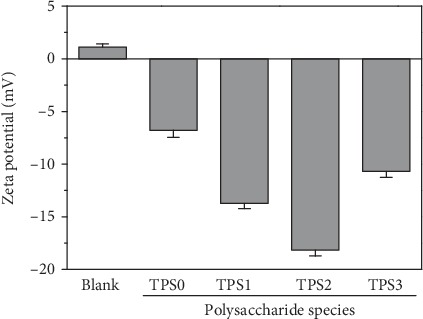
Zeta potential of CaOx crystals induced by TPSs with different molecular weights. *c*(TPS) = 0.1 g/L; crystallization time: 2 h.

The changes in *ζ* of the CaOx crystals generated under the regulation of TPSs with different molecular weights are shown in Figure 6. The *ζ* values of the crystals without polysaccharide were 1.12 mV. However, the absolute value of *ζ* in CaOx crystals in the presence of TPSs increased, thereby indicating that the charge density on the crystal surface increased, the repulsive force between crystals increased, and the degree of aggregation between crystals decreased. Given that the absolute *ζ* value of the crystal was the largest (18.17 mV) in the presence of TPS2, TPS2 had the best performance in inhibiting crystal aggregation.

### 3.6. Protective Effects of TPs on HK-2 Cells

#### 3.6.1. Cytotoxicity of TPSs

**Figure 7:**
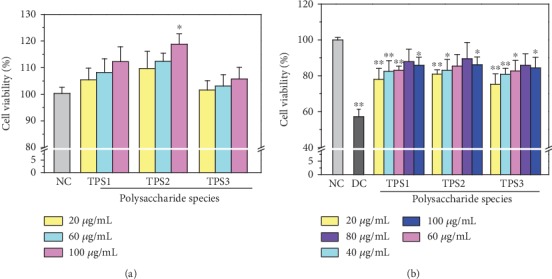
(a) Cytotoxicity detection of TPSs with different molecular weights on HK-2 cells. (b) Cell viability changes of HK-2 cell before and after TPSs protection. NC: normal control; DC: damaged control; oxalate damage concentration: 2.8 mmol/L; damage time: 3.5 h; protective time: 12 h.

Given that TPS0 had a weak ability to regulate CaOx crystallization, only TPS1, TPS2, and TPS3 were selected for subsequent cell experiments. TPS1, TPS2, and TPS3 showed no cytotoxicity toward HK-2 cells and had a proliferative effect (100.31%–118.78%, Figure 7(a).

#### 3.6.2. TPS Protection Increased Antioxidative Damage Ability of HK-2 cells

As shown in Figure 7(b), the cell viability of normal HK-2 cells decreased from 100% to 57.16% after being injured by 2.8 mmol/L oxalic acid. The HK-2 cells were preprotected with TPSs at 20, 40, 60, 80, and 100 *μ*g/mL for 12 h, and then, 2.8 mmol/L oxalic acid was added. The viability of the cells (75.29%–89.45%) was higher than that of the injured group (57.16%). These results indicated that three TPSs can increase cell resistance to oxidative damage caused by oxalic acid, and the effect was the best when the concentration was 80 *μ*g/mL.

#### 3.6.3. Release of Lactate Dehydrogenase (LDH) Reduced after Protection by TPSs

**Figure 8:**
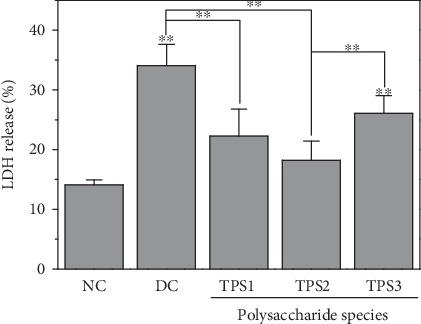
LDH release from HK-2 cells before and after TPS protection. *c*(TPS) = 80 *μ*g/mL. NC: normal control, DC: damaged control; oxalate damage concentration: 2.8 mmol/L; damage time: 3.5 h; protective time: 12 h. Compared with NC group, ^∗^*p* < 0.05, ^∗∗^*p* < 0.01.

LDH is a cytoplasmic enzyme that stably exists in the cytoplasm. When the cell membrane is damaged, LDH is released outside the cell. Therefore, the degree of cell membrane damage can be judged by detecting LDH release.  Figure 8 shows the LDH detection results of normal, injured, and protected cells. Compared with the injured cells, the LDH release of the preprotected cells decreased, thereby indicating that the degree of cell membrane damage decreased, and the protective ability of TPS2 was greater than those of TPS1 and TPS3.

#### 3.6.4. Changes in Cell Morphology after Protection by TPSs

**Figure 9:**
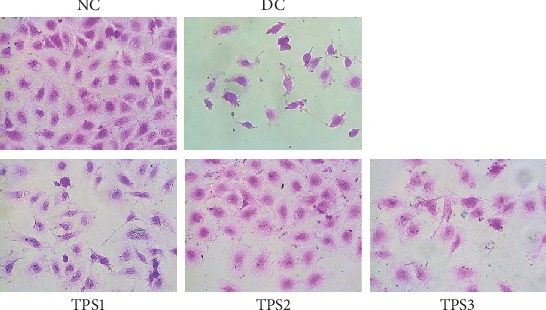
Morphological changes of HK-2 cells before and after TPS protection. *c*(TPS) = 80 *μ*g/mL. The experimental conditions are the same as Figure 8.

Figure 9 shows the changes in cell morphology before and after TPS protection. The junctions between normal HK-2 cells were tight, and the cells were plump. However, in the injured group, cell density decreased, cell morphology was irregular, and the volume of some cells decreased. After preprotection by TPSs with different molecular weights, the degree of damage of oxalic acid to cell morphology decreased. In particular, the cell morphology of the TPS2 protection group was close to normal cells.

#### 3.6.5. Changes in Intracellular Reactive Oxygen Species (ROS) after Protection by TPSs

**Figure 10:**
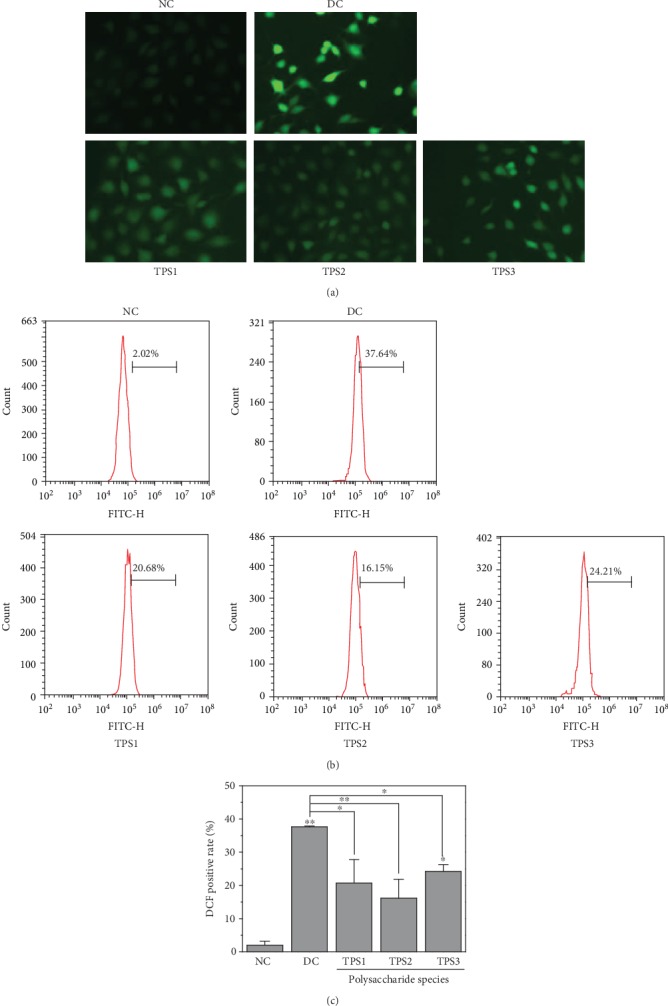
ROS changes in HK-2 cells before and after TPS protection. (a) Fluorescence microscopy images. (b) Flow cytometry quantitative analysis of fluorescence intensity. (c) Quantitative fluorescence intensity of ROS. *c*(TPS) = 80 *μ*g/mL. The experimental conditions and statistical significance are the same as Figure 8.

Figure 10 shows the changes in the ROS of cells after protecting the three TPSs. The green fluorescence of the cells in the normal group was the weakest (Figure 10(a)), thereby indicating that the ROS level was the lowest. However, ROS was highest in the injured group. After TPS preprotection, the ROS level of the cells was lower than that of the injured group.

Figure 10(b) is a result of quantitative detection of the fluorescence intensity of the ROS generated by cells. Compared with the normal group (2.02%), the fluorescence intensity of the injured cells (37.64%) was the strongest; that is, the ROS level of injured cells was the highest. After being preprotected by TPSs, the fluorescence intensity of the cells decreased at different degrees (16.15%–24.21%, Figure 10(c)). These results indicated that TPSs can reduce ROS generation in cells; that is, TPSs reduced the oxidative damage of cells. The degree of reduction was remarkably significant in the TPS2 group (16.15%).

## 4. Discussion

**Figure 11:**
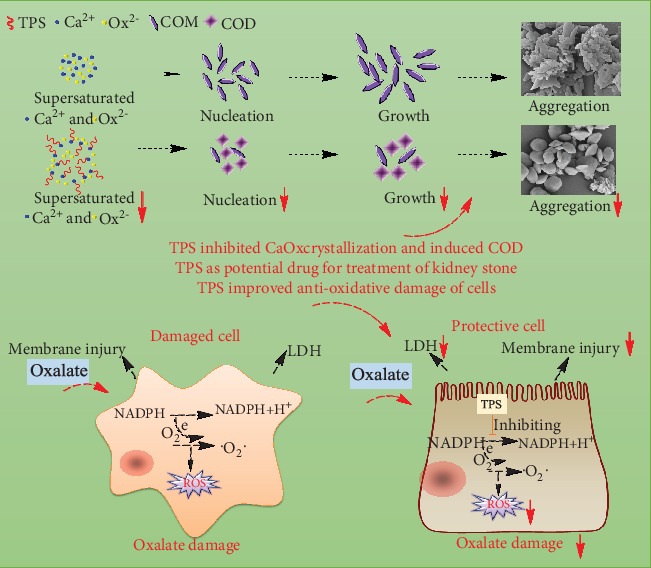
Mechanism diagram of TPSs regulating CaOx crystal growth and protecting HK-2 cells from oxidative damage.

The formation of kidney stones involves the nucleation, growth, and aggregation of CaOx crystals, as well as the adhesion of crystals to renal epithelial cells. Many acidic molecules in urine, such as citric acid, GAGs, and osteopontin, can inhibit the processes above. TPSs containing abundant –COOH groups have a chemical structure similar to that of a GAG [26]. Thus, TPSs are potentially effective drugs for inhibiting CaOx kidney stones. This study is aimed at investigating the protective effects of TPSs with different molecular weights on HK-2 cells and TPS regulation on CaOx crystallization in vitro (Figure 11).

### 4.1. TPSs Induced COD Formation

All the FT-IR, XRD, and SEM measurements showed that three degraded TPSs, namely, TPS1, TPS2, and TPS3, can inhibit COM growth and promote COD formation. The induced COD percentage increased with TPS concentration, and TPS2 with a moderate molecular weight had the best effect.

TPSs promote COD formation because TPSs containing acidic groups can adsorb large amounts of Ca^2+^ ions in the solution by electrostatic interaction, which increases the [Ca^2+^]/[Ox^2−^] ratio in the partial area. Excessive Ca^2+^ ions tend to promote the formation of COD crystals, and excessive Ox^2−^ ions promote the formation of COM crystals [27]. Therefore, COD crystals are easily formed in the presence of TPSs. Given that the polysaccharide surface was enriched with positively charged Ca ions, a high-energy interface was also formed. At the same time, the degree of freedom of the adsorbed Ca ions decreased, and the energy state increased. The high-energy interface and state both promoted the COD formation [28].

Given that the adhesion force between COD crystal and renal cell membrane surface is smaller than COM [29, 30], crystals are relatively easy to be excreted out of the body with urine. Reducing the formation of COM is beneficial to inhibit kidney stone formation. Thus, TPS2 with a moderate molecular weight may have an improved effect on inhibiting urinary stones.

### 4.2. TPSs Inhibited CaOx Crystal Nucleation and Aggregation

TPSs can effectively inhibit CaOx crystal nucleation and aggregation, and TPS2 had the best effect because when TPSs, which is rich in –COOH groups, formed soluble complexes with Ca^2+^ ions, the degree of supersaturation of CaOx solution decreased and thereby reduced the *S*_N_ of the crystallites. The adsorption of TPSs on crystal caused negative *ζ* on the crystal surface (Figure 6), thereby increasing the repulsion between the crystals, which inhibited crystal aggregation.

### 4.3. Degraded TPSs Had Protective Effects on HK-2 Cells

After oxalic acid caused oxidative damage to HK-2 cells, the cell viability decreased (Figure 7), the cell membrane was destroyed, LDH release increased (Figure 8), the cell volume decreased, and tight connections between cells were destroyed (Figure 9). However, the preprotected cells had a strong ability of antioxidative damage. After the cells were injured, excessive ROS was generated by affecting NADPH oxidase activity. ROS accumulation in cells will cause oxidative stress reaction [31]. TPS preprotection can significantly reduce the ROS level of cells (Figure 10), thereby improving the antioxidant capacity of cells.

### 4.4. Reason for Best Inhibitory Effect of TPS2 with Moderate Molecular Weight

Many factors, such as molecular weight of polysaccharide, acid group content in polysaccharide, glycosidic bond, monosaccharide type, and degree of branching, affect the biological activity of polysaccharide [9], among which molecular weight is one of the most important factors.

1. The biological activity of polysaccharide with a large molecular weight decreased. The reason may be due to the following: (a) polysaccharide with an excessive molecular weight has a strong H bond effect and compact structure, which hinders the formation of unfolded structures; (b) polysaccharides have branched chains, large steric hindrance, and large volume [32]; (c) high-molecular-weight polysaccharides exhibit low water solubility, polysaccharide exposed to less active groups in solution than low-molecular-weight polysaccharide, and the interactions between polysaccharides and Ca^2+^ are reduced. Therefore, polysaccharides with an excessive molecular weight cannot effectively inhibit the nucleation and aggregation of crystals and regulate the crystal phase of calcium oxalate. High-molecular-weight polysaccharides feature a large molecular volume which cannot easily cross the cell membrane and enter the cells to exert their biological activity. Sheng and Sun [33] showed that polysaccharides with a low molecular weight (1.45–6.4 kDa) have stronger antioxidant activities than those with larger molecular weights (16.89 kDa).

The regulation ability of TPS on CaOx crystals is affected by both its carboxyl group (–COOH) content and molecular weight. TPS2 with a moderate molecular weight and rich in carboxyl group is more conducive to promoting the formation of COD crystals. Although TPS0 (highest MW) and TPS3 (lowest MW) are quite similar with respect to the number of acidic groups they contain (11.2% vs 11.0%), TPS0 did not induce COD formation, while TPS3 induced 31% COD formation. This is because large-molecular-weight polysaccharides have large steric hindrance and less exposed active groups; these factors hinder the interaction between high-molecular-weight TPS0 and Ca^2+^, resulting in a decrease in the accumulating ability of TPS0 to Ca^2+^, and it cannot effectively increase the local area [Ca^2+^]/[Ox^2-^] ratio, so TPS0 did not induce COD formation. Although the number of acidic groups of TPS3 is similar to TPS0, the molecular weight of TPS3 (2.31 kDa) is much smaller than that of TPS0 (10.88 kDa). Therefore, the above factors that hinder the biological activity for TPS0 are obviously reduced for TPS3.

(2) The biological activity of the polysaccharides with an extremely small molecular weight decreases because polysaccharide with a small molecular weight has a relatively short polysaccharide chain, and the H bond between the polysaccharide chains is weak, which cannot form a complete triple helix polymerization structure with biological activity [34]. The polysaccharide cannot exert its biological activity [35]. For example, Deng et al. [36] showed that the antitumor activity of *Lycium barbarum* polysaccharide with a molecular weight of 40 kDa on mouse liver cancer cells is better than that of 3 kDa
(3) Only polysaccharide with a moderate molecular weight had the strongest biological activity. Moderate-molecular-weight TPS2 not only had enough space positions to form a complete polymerization structure but also broke the highly compact molecular conformation of the high-molecular-weight polysaccharide and formed unfolded structures, thereby exposing inner functional groups (e.g., −COOH) in polysaccharides. The polysaccharide with a moderate molecular weight has a high degree of freedom and small steric hindrance. The –COOH content (12.7%) in TPS2 was slightly higher than those of the three other TPSs (11.0%–12.3%).

The factors above are the reasons for the significant increase in the absolute *ζ* value on the crystal surface that was adsorbed with TPS2 (Figure 6) and increase in the repulsive force between crystals. Given that TPS2 can complex additional Ca^2+^ ions, it has a good inhibiting effect on crystal nucleation and aggregation and can easily enter cells and protect cells from oxidative damage [37]. You et al. [38] showed that *Lentinus edodes* polysaccharides with a moderate molecular weight (306 kDa) has the strongest protective effect on mouse cardiomyocytes against oxidative stress induced by d-galactose compared with those with extremely large (605.0 kDa) and small molecular weights (25.5 kDa).

## 5. Conclusions

The three degraded TPSs, namely, TPS1, TPS2, and TPS3, have inhibitory effects on the nucleation and aggregation of the CaOx crystals, inhibit COM growth, and induce COD formation. TPSs can protect HK-2 cells from oxidative damage, of which TPS2 with moderate molecular weight has the best effect. Therefore, TPSs, especially TPS2, may prevent the formation of kidney stones.

## Figures and Tables

**Table 1:** Carboxyl content of TPSs with different molecular weights and CaOx crystal phase difference induced by 0.1 g/L of TPS.

TPS	Molecular weight (kDa)	–COOH content (%)	*c*(TPS) (g/L)	COM content (%)	COD content (%)
Blank	0	—	0	100	0
TPS0	10.88	11.2	0.1	100	0
TPS1	8.16	12.3	0.1	27	73
TPS2	4.82	12.7	0.1	20	80
TPS3	2.31	11.0	0.1	69	31

**Table 2:** Infrared characteristic absorption peaks of CaOx crystals formed in the presence of different concentrations of TPSs.

TPSs	c(TPS) (g/L)	COD (%)	*a*_*νs*_(COO−) (cm^−1^)	*ν*_*as*_(*COO*−) (cm^−1^)	*ν*_*s*_(*COO*−) (cm^−1^)	COM (cm^−1^)	COD (cm^−1^)	COM (cm^−1^)	COM (cm^−1^)	COM (cm^−1^)	COD (cm^−1^)
Blank	0	0		1617	1325	942		879	781	657	

TPS0	0.1	0		1617	1329	950		878	786	651	
	0.15	0		1618	1329	950		878	786	654	
	0.3	0		1619	1332	953		884	786	655	
	0.5	0		1621	1332	953		883	786	666	
	0.8	0		1627	1334	959		888	792	673	

TPS1	0.1	73	1639		1323		912		786		620
	0.15	84.8	1640		1333		912		786		621
	0.3	100	1645		1334		912				625
	0.5	100	1647		1334		916				625
	0.8	100	1650		1340		917				620

TPS2	0.1	80	1639		1323		917		769		620
	0.15	87	1645		1334		923		780		625
	0.3	100	1646		1337		917				625
	0.5	100	1647		1340		917				616
	0.8	100	1651		1342		918				616

TPS3	0.1	31		1620	1321			885	785	670	618
	0.15	79		1629	1327		912		790		622
	0.3	100	1639		1332		915				616
	0.5	100	1643		1334		916				612
	0.8	100	1646		1335		918				612

COM				1621	1325	947		885	780	663	

COD			1647		1326		916				618

**Table 3:** Inhibitory effect of nucleation rate and aggregation rate of CaOx crystals by TPSs.

	Blank	TPS0	TPS1	TPS2	TPS3
*t*_max_ (s)	540	630	750	840.	660
*S*_N_ (×10^−5^/s)	17.1 ± 3.12	15.2 ± 3.18	7.41 ± 0.29	4.22 ± 0.98	8.05 ± 0.51
*S*_A_ (×10^−5^/s)	9.31 ± 1.97	8.58 ± 0.73	7.23 ± 0.91	4.88 ± 0.49	7.3 ± 0.64

Note: *t*_max_ (s): the moment at which maximum absorbance; *S*_N_: rate of crystal nucleation; *S*_A_: rate of crystal aggregation.

## Data Availability

All the data supporting the results were shown in the paper, and can be applicable from the corresponding author.
